# Supplementary figures and images for: Population structure of wild soybean (Glycine soja) based on SLAF-seq have implications for its conservation
Source: PeerJ. 2023 Nov 8;11:e16415. doi: 10.7717/peerj.16415 (PMC10638924; doi:10.7717/peerj.16415)

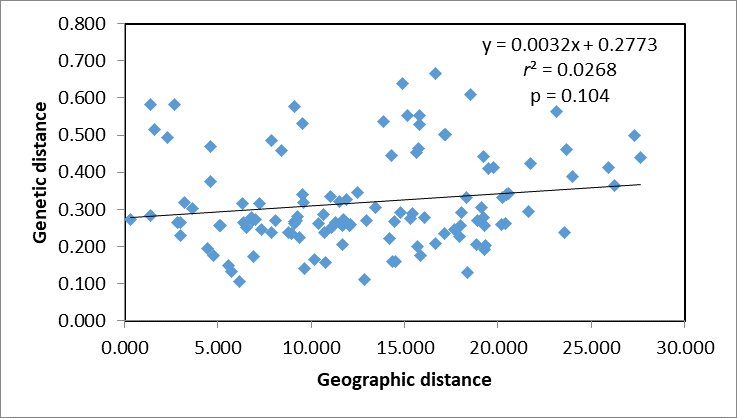

Supplement: Supplemental Information 1 [file peerj-11-16415-s001.png]
