# Supplementary material for: Population structure of wild soybean (Glycine soja) based on SLAF-seq have implications for its conservation
Source: PeerJ. 2023 Nov 8;11:e16415. doi: 10.7717/peerj.16415 (PMC10638924; doi:10.7717/peerj.16415)
Supplement: Supplemental Information 2 [file peerj-11-16415-s002.docx]

Table S1 Information for the raw and quality filtered sequence reads obtained by SLAF-seq of 147 wild soybean individuals

| Data No. | Sample ID. | Population ID. | Clean Reads No. | Total nucleotides (Mb) | Tag No. | Coverage (X) | GC Percentage (%) | Q30 Percentage (%) | accession numbers |
| --- | --- | --- | --- | --- | --- | --- | --- | --- | --- |
| 1 | DQ-1 | DQ | 3810972 | 762.19 | 170923 | 11 | 40.85 | 91.02 | SRR17650031 |
| 2 | DQ-2 | DQ | 2966660 | 593.33 | 196971 | 9 | 40.15 | 90.18 | SRR17650032 |
| 3 | DQ-3 | DQ | 3633741 | 726.75 | 158578 | 11 | 41.14 | 91.44 | SRR17650033 |
| 4 | WH-1 | WH | 3280852 | 656.17 | 217823 | 8 | 40.23 | 91.71 | SRR17650034 |
| 5 | WH-2 | WH | 2790421 | 558.08 | 179663 | 8 | 40.68 | 90.17 | SRR17650035 |
| 6 | WH-3 | WH | 2266715 | 453.34 | 146081 | 8 | 40.79 | 90.67 | SRR17650036 |
| 7 | WH-4 | WH | 2677159 | 535.43 | 171603 | 8 | 41.1 | 90.4 | SRR17650037 |
| 8 | WH-5 | WH | 3022832 | 604.57 | 167544 | 12 | 40.12 | 88.84 | SRR17650038 |
| 9 | WH-6 | WH | 2367623 | 473.52 | 150113 | 9 | 40.83 | 89.79 | SRR17650039 |
| 10 | WH-7 | WH | 2376274 | 475.25 | 156729 | 9 | 40.5 | 89.89 | SRR17650040 |
| 11 | WH-8 | WH | 3859838 | 771.97 | 211599 | 12 | 39.62 | 90.05 | SRR17650041 |
| 12 | WH-9 | WH | 3137074 | 627.41 | 195505 | 10 | 39.97 | 90.1 | SRR17650042 |
| 13 | WN-1 | WN | 3363505 | 672.70 | 197439 | 11 | 39.41 | 89.68 | SRR17650043 |
| 14 | WN-2 | WN | 7610035 | 1522.01 | 227189 | 25 | 38.56 | 87.79 | SRR17650044 |
| 15 | WN-3 | WN | 2837792 | 567.56 | 193399 | 9 | 39.82 | 90.7 | SRR17650045 |
| 16 | WN-4 | WN | 3262046 | 652.41 | 219200 | 9 | 39.81 | 90.55 | SRR17650046 |
| 17 | WN-5 | WN | 2664751 | 532.95 | 170903 | 9 | 39.98 | 89.04 | SRR17650047 |
| 18 | WN-6 | WN | 3314426 | 662.89 | 206973 | 10 | 39.11 | 90.74 | SRR17650048 |
| 19 | WN-7 | WN | 2745891 | 549.18 | 184008 | 8 | 39.67 | 90.26 | SRR17650049 |
| 20 | WN-8 | WN | 3418666 | 683.73 | 199255 | 10 | 39.49 | 89.2 | SRR17650050 |
| 21 | WN-9 | WN | 2964749 | 592.95 | 187308 | 10 | 39.91 | 90.21 | SRR17650051 |
| 22 | CC-1 | CC | 3403868 | 680.77 | 215919 | 9 | 39.93 | 89.99 | SRR17650052 |
| 23 | CC-2 | CC | 2847626 | 569.53 | 184471 | 9 | 39.87 | 89.38 | SRR17650053 |
| 24 | CC-3 | CC | 3360684 | 672.14 | 206087 | 10 | 39.8 | 90.12 | SRR17650054 |
| 25 | CC-4 | CC | 3421978 | 684.40 | 228594 | 9 | 40.17 | 90.95 | SRR17650055 |
| 26 | CC-5 | CC | 3345981 | 669.20 | 229710 | 8 | 40.16 | 91.37 | SRR17650056 |
| 27 | CC-6 | CC | 2713894 | 542.78 | 177535 | 8 | 40.81 | 91.31 | SRR17650057 |
| 28 | CC-7 | CC | 2933072 | 586.61 | 201921 | 8 | 40.47 | 91.08 | SRR17650058 |
| 29 | CC-8 | CC | 2764581 | 552.92 | 184890 | 8 | 40.07 | 90.58 | SRR17650059 |
| 30 | CC-9 | CC | 2915078 | 583.02 | 198600 | 8 | 40.03 | 91.43 | SRR17650060 |
| 31 | CC-10 | CC | 3008949 | 601.79 | 207178 | 8 | 40.33 | 91.6 | SRR17650061 |
| 32 | JN-1 | JN | 3246425 | 649.29 | 229092 | 8 | 41.41 | 91.43 | SRR17650062 |
| 33 | JN-2 | JN | 3236386 | 647.28 | 221394 | 8 | 40.55 | 91.21 | SRR17650063 |
| 34 | JN-3 | JN | 3070257 | 614.05 | 217935 | 8 | 40.76 | 91.46 | SRR17650064 |
| 35 | JN-4 | JN | 3002420 | 600.48 | 198416 | 9 | 40.48 | 89.02 | SRR17650065 |
| 36 | JN-5 | JN | 2889476 | 577.90 | 192285 | 9 | 40.32 | 90.71 | SRR17650066 |
| 37 | JN-6 | JN | 2993347 | 598.67 | 202102 | 9 | 40.08 | 91.09 | SRR17650067 |
| 38 | JN-7 | JN | 2902805 | 580.56 | 195278 | 8 | 40.32 | 91.88 | SRR17650068 |
| 39 | JN-8 | JN | 2859179 | 571.84 | 198939 | 8 | 40.21 | 91.19 | SRR17650069 |
| 40 | JN-9 | JN | 3171380 | 634.28 | 215197 | 9 | 40.43 | 90.66 | SRR17650070 |
| 41 | JN-10 | JN | 2865375 | 573.08 | 195701 | 8 | 40.45 | 90.94 | SRR17650071 |
| 42 | QQHE-1 | QQHE | 2707632 | 541.53 | 188829 | 8 | 40.22 | 90.76 | SRR17650072 |
| 43 | QQHE-2 | QQHE | 3022336 | 604.47 | 198682 | 9 | 40.18 | 90.75 | SRR17650073 |
| 44 | QQHE-3 | QQHE | 2808376 | 561.68 | 182987 | 9 | 39.98 | 90.17 | SRR17650074 |
| 45 | QQHE-4 | QQHE | 2895687 | 579.14 | 192251 | 8 | 40.67 | 90.67 | SRR17650075 |
| 46 | QQHE-5 | QQHE | 3171382 | 634.28 | 206198 | 9 | 39.86 | 91.26 | SRR17650076 |
| 47 | QQHE-6 | QQHE | 2764120 | 552.82 | 183775 | 8 | 40.55 | 91.11 | SRR17650077 |
| 48 | QQHE-7 | QQHE | 3807845 | 761.57 | 200929 | 12 | 39.49 | 89.4 | SRR17650078 |
| 49 | QQHE-8 | QQHE | 3780103 | 756.02 | 217754 | 11 | 39.89 | 89.89 | SRR17650079 |
| 50 | QQHE-9 | QQHE | 4254015 | 850.80 | 231536 | 12 | 38.52 | 89.08 | SRR17650080 |
| 51 | QQHE-10 | QQHE | 3433292 | 686.66 | 197027 | 12 | 38.28 | 89.14 | SRR17650081 |
| 52 | YW-1 | YW | 3377384 | 675.48 | 192530 | 11 | 39.79 | 90.92 | SRR17650082 |
| 53 | YW-2 | YW | 4389702 | 877.94 | 226373 | 13 | 39.49 | 89.49 | SRR17650083 |
| 54 | YW-3 | YW | 3981451 | 796.29 | 233045 | 12 | 38.4 | 89.87 | SRR17650084 |
| 55 | YW-4 | YW | 3877244 | 775.45 | 206737 | 12 | 39.41 | 90.25 | SRR17650085 |
| 56 | YW-5 | YW | 3095320 | 619.06 | 182447 | 11 | 39.54 | 89.7 | SRR17650086 |
| 57 | YW-6 | YW | 3416729 | 683.35 | 190983 | 12 | 39.33 | 89.23 | SRR17650087 |
| 58 | YW-7 | YW | 3900619 | 780.12 | 209767 | 12 | 39.23 | 89.08 | SRR17650088 |
| 59 | YW-8 | YW | 4648633 | 929.73 | 237990 | 13 | 38.88 | 89.59 | SRR17650089 |
| 60 | YW-9 | YW | 4093526 | 818.71 | 215393 | 13 | 39.07 | 89.81 | SRR17650090 |
| 61 | YW-10 | YW | 4864071 | 972.81 | 247347 | 14 | 38.7 | 89.37 | SRR17650091 |
| 62 | SY-1 | SY | 4117299 | 823.46 | 212454 | 13 | 38.83 | 89.99 | SRR17650092 |
| 63 | SY-2 | SY | 4484971 | 896.99 | 228247 | 14 | 38.98 | 89.16 | SRR17650093 |
| 64 | SY-3 | SY | 4751729 | 950.35 | 237508 | 14 | 38.88 | 89.9 | SRR17650094 |
| 65 | SY-4 | SY | 4647559 | 929.51 | 237393 | 13 | 38.68 | 90.28 | SRR17650095 |
| 66 | SY-5 | SY | 3069880 | 613.98 | 176500 | 11 | 38.99 | 89.95 | SRR17650096 |
| 67 | SY-6 | SY | 5939829 | 1187.97 | 223101 | 17 | 39.5 | 89.72 | SRR17650097 |
| 68 | SY-7 | SY | 4197432 | 839.49 | 226907 | 12 | 38.38 | 89.36 | SRR17650098 |
| 69 | SY-8 | SY | 3775707 | 755.14 | 201346 | 12 | 39.75 | 89.32 | SRR17650099 |
| 70 | SY-9 | SY | 5541120 | 1108.22 | 248913 | 15 | 39.96 | 88.84 | SRR17650100 |
| 71 | SY-10 | SY | 4304171 | 860.83 | 219837 | 13 | 38.86 | 89.42 | SRR17650101 |
| 72 | TJ-1 | TJ | 4354647 | 870.93 | 221689 | 13 | 38.97 | 88.32 | SRR17650102 |
| 73 | TJ-2 | TJ | 4034481 | 806.90 | 216013 | 12 | 39.24 | 87.42 | SRR17650103 |
| 74 | TJ-3 | TJ | 5319536 | 1063.91 | 223815 | 16 | 38.23 | 89.21 | SRR17650104 |
| 75 | TJ-4 | TJ | 3056043 | 611.21 | 186434 | 11 | 38.49 | 89.89 | SRR17650105 |
| 76 | TJ-5 | TJ | 4541815 | 908.36 | 244913 | 12 | 39.04 | 90.02 | SRR17650106 |
| 77 | TJ-6 | TJ | 4155098 | 831.02 | 207047 | 13 | 39.85 | 89.41 | SRR17650107 |
| 78 | TJ-7 | TJ | 6878471 | 1375.69 | 223479 | 22 | 38.48 | 88.69 | SRR17650108 |
| 79 | TJ-8 | TJ | 3775767 | 755.15 | 224422 | 12 | 38.09 | 89.6 | SRR17650109 |
| 80 | TJ-9 | TJ | 5453188 | 1090.64 | 212405 | 17 | 38.58 | 88.49 | SRR17650110 |
| 81 | TJ-10 | TJ | 3823582 | 764.72 | 241282 | 11 | 38.98 | 89.35 | SRR17650111 |
| 82 | HH-1 | HH | 4136061 | 827.21 | 199864 | 13 | 39.42 | 88.58 | SRR17650112 |
| 83 | HH-2 | HH | 6254400 | 1250.88 | 226848 | 18 | 39.16 | 88.18 | SRR17650113 |
| 84 | HH-3 | HH | 5710363 | 1142.07 | 197947 | 18 | 39.88 | 88.26 | SRR17650114 |
| 85 | HH-4 | HH | 3093203 | 618.64 | 182123 | 11 | 39.76 | 88.9 | SRR17650115 |
| 86 | HH-5 | HH | 3619730 | 723.95 | 204229 | 12 | 39.23 | 88.74 | SRR17650116 |
| 87 | HH-6 | HH | 4195214 | 839.04 | 211399 | 13 | 40 | 88.83 | SRR17650117 |
| 88 | HH-7 | HH | 3403029 | 680.61 | 182873 | 12 | 40.01 | 89.54 | SRR17650118 |
| 89 | HH-8 | HH | 3276232 | 655.25 | 181129 | 11 | 40.1 | 89.05 | SRR17650119 |
| 90 | HH-9 | HH | 2947604 | 589.52 | 161415 | 11 | 40.01 | 89.74 | SRR17650120 |
| 91 | HH-10 | HH | 4010915 | 802.18 | 218453 | 12 | 39.35 | 88.78 | SRR17650121 |
| 92 | HEB-1 | HEB | 4595818 | 919.16 | 241391 | 13 | 38.66 | 88.49 | SRR17650122 |
| 93 | HEB-2 | HEB | 3650112 | 730.02 | 205693 | 12 | 38.72 | 89.31 | SRR17650123 |
| 94 | HEB-3 | HEB | 4367435 | 873.49 | 219927 | 13 | 39.21 | 88.64 | SRR17650124 |
| 95 | HEB-4 | HEB | 4400563 | 880.11 | 233266 | 13 | 38.46 | 87.45 | SRR17650125 |
| 96 | HEB-5 | HEB | 4662464 | 932.49 | 244035 | 13 | 38.48 | 88.31 | SRR17650126 |
| 97 | HEB-6 | HEB | 3869378 | 773.88 | 190020 | 13 | 39.47 | 88.93 | SRR17650127 |
| 98 | HEB-7 | HEB | 3929800 | 785.96 | 207802 | 13 | 38.96 | 88.83 | SRR17650128 |
| 99 | HEB-8 | HEB | 3778585 | 755.72 | 203523 | 12 | 38.68 | 88.82 | SRR17650129 |
| 100 | HEB-9 | HEB | 4388294 | 877.66 | 238840 | 12 | 38.27 | 88.88 | SRR17650130 |
| 101 | HEB-10 | HEB | 3550185 | 710.04 | 195717 | 12 | 38.51 | 88 | SRR17650131 |
| 102 | NJ-1 | NJ | 5220085 | 1044.02 | 215481 | 16 | 38.37 | 88.81 | SRR17650132 |
| 103 | NJ-2 | NJ | 7726419 | 1545.28 | 229852 | 22 | 38.64 | 88.27 | SRR17650133 |
| 104 | NJ-3 | NJ | 5686366 | 1137.27 | 229672 | 17 | 38.72 | 87.9 | SRR17650134 |
| 105 | NJ-4 | NJ | 4936746 | 987.35 | 217095 | 15 | 38.53 | 88.59 | SRR17650135 |
| 106 | NJ-5 | NJ | 3746457 | 749.29 | 201806 | 12 | 39.25 | 88.33 | SRR17650136 |
| 107 | NJ-6 | NJ | 2838858 | 567.77 | 186152 | 10 | 37.88 | 89.4 | SRR17650137 |
| 108 | NJ-7 | NJ | 2922183 | 584.44 | 190694 | 10 | 38.35 | 88.64 | SRR17650138 |
| 109 | NJ-8 | NJ | 3190389 | 638.08 | 193106 | 11 | 38.96 | 87.39 | SRR17650139 |
| 110 | NJ-9 | NJ | 3543429 | 708.69 | 203941 | 11 | 38.9 | 88.65 | SRR17650140 |
| 111 | NJ-10 | NJ | 5981356 | 1196.27 | 247463 | 16 | 38.26 | 88.03 | SRR17650141 |
| 112 | KO-1 | KO | 3360877 | 672.18 | 193122 | 11 | 40.84 | 90.86 | SRR17650142 |
| 113 | KO-2 | KO | 4055920 | 811.18 | 228277 | 12 | 39.71 | 89.21 | SRR17650143 |
| 114 | KO-3 | KO | 4347376 | 869.48 | 231419 | 13 | 39.68 | 90.48 | SRR17650144 |
| 115 | KO-4 | KO | 4477468 | 895.49 | 240405 | 12 | 40 | 89.93 | SRR17650145 |
| 116 | KO-5 | KO | 4263580 | 852.72 | 233567 | 13 | 39.3 | 89.66 | SRR17650146 |
| 117 | KO-6 | KO | 4245636 | 849.13 | 228282 | 13 | 39.52 | 89.77 | SRR17650147 |
| 118 | KO-7 | KO | 3668013 | 733.60 | 207289 | 11 | 40.68 | 90.01 | SRR17650148 |
| 119 | KO-8 | KO | 3010659 | 602.13 | 175670 | 10 | 41.23 | 90.08 | SRR17650149 |
| 120 | KR-1 | KR | 3893515 | 778.70 | 222520 | 11 | 40.13 | 90 | SRR17650150 |
| 121 | KR-2 | KR | 3830281 | 766.06 | 207300 | 12 | 40 | 90.3 | SRR17650151 |
| 122 | KR-3 | KR | 4321878 | 864.38 | 237582 | 12 | 40.17 | 90.61 | SRR17650152 |
| 123 | KR-4 | KR | 3768913 | 753.78 | 211223 | 11 | 40.57 | 90.81 | SRR17650153 |
| 124 | KR-5 | KR | 3899929 | 779.99 | 217865 | 12 | 39.63 | 89.82 | SRR17650154 |
| 125 | KR-6 | KR | 3914278 | 782.86 | 211131 | 13 | 39.49 | 90.07 | SRR17650155 |
| 126 | JK-1 | JK | 3228815 | 645.76 | 178143 | 11 | 40.83 | 90.25 | SRR17650156 |
| 127 | JK-2 | JK | 5342161 | 1068.43 | 202610 | 17 | 40.28 | 90.4 | SRR17650157 |
| 128 | JK-3 | JK | 3382619 | 676.52 | 183147 | 12 | 39.72 | 90.26 | SRR17650158 |
| 129 | JK-4 | JK | 3034376 | 606.88 | 161421 | 12 | 40.45 | 90.63 | SRR17650159 |
| 130 | JK-5 | JK | 3772127 | 754.43 | 213501 | 11 | 40.44 | 91.21 | SRR17650160 |
| 131 | JK-6 | JK | 3463463 | 692.69 | 200882 | 11 | 40.04 | 90.52 | SRR17650161 |
| 132 | JK-7 | JK | 2950809 | 590.16 | 166452 | 11 | 40.45 | 90.73 | SRR17650162 |
| 133 | JK-8 | JK | 4316278 | 863.26 | 210646 | 14 | 39.74 | 90.38 | SRR17650163 |
| 134 | JK-9 | JK | 3722006 | 744.40 | 202232 | 11 | 40.46 | 90.1 | SRR17650164 |
| 135 | JK-10 | JK | 3467758 | 693.55 | 200661 | 11 | 40.21 | 90.93 | SRR17650165 |
| 136 | JK-11 | JK | 3270470 | 654.09 | 192856 | 11 | 40.41 | 90.38 | SRR17650166 |
| 137 | JK-12 | JK | 2820569 | 564.11 | 172644 | 10 | 40.32 | 90.36 | SRR17650167 |
| 138 | JT-1 | JT | 3206556 | 641.31 | 183558 | 11 | 40.19 | 89.76 | SRR17650168 |
| 139 | JT-2 | JT | 3255622 | 651.12 | 188552 | 11 | 40.1 | 90.34 | SRR17650169 |
| 140 | JT-3 | JT | 3699285 | 739.86 | 210071 | 11 | 40.01 | 90.53 | SRR17650170 |
| 141 | JT-4 | JT | 11010066 | 2202.01 | 194550 | 34 | 39.31 | 90.2 | SRR17650171 |
| 142 | JT-5 | JT | 3048089 | 609.62 | 167289 | 11 | 40.45 | 90.7 | SRR17650172 |
| 143 | JT-6 | JT | 5482684 | 1096.54 | 122838 | 18 | 40.77 | 90.03 | SRR17650173 |
| 144 | JT-7 | JT | 3840969 | 768.19 | 153232 | 16 | 40.08 | 90 | SRR17650174 |
| 145 | JT-8 | JT | 7064413 | 1412.88 | 125847 | 23 | 40.75 | 90.39 | SRR17650175 |
| 146 | JT-9 | JT | 5802576 | 1160.52 | 132704 | 21 | 40.4 | 90.16 | SRR17650176 |
| 147 | JT-10 | JT | 3645756 | 729.15 | 154013 | 15 | 40.22 | 89.85 | SRR17650177 |
| Average |  |  | 3859551.3 | 771.9 | 202663.9 | 11.9 | 39.7 | 89.8 |  |
